# The Continuing Growth of Global Cooperation Networks in Research: A Conundrum for National Governments

**DOI:** 10.1371/journal.pone.0131816

**Published:** 2015-07-21

**Authors:** Caroline S. Wagner, Han Woo Park, Loet Leydesdorff

**Affiliations:** 1 Milton & Roslyn Wolf Chair in International Affairs, John Glenn School of Public Affairs, and Director, Battelle Center for Science & Technology Policy, The Ohio State University, Columbus, Ohio, United States of America; 2 Department of Media & Communication, YeungNam University, Dae-dong, Gyeongsan-si, Gyeongsangbuk-do, South Korea; 3 Amsterdam School of Communications Research, University of Amsterdam, Amsterdam, The Netherlands; Katholieke Universiteit Leuven, BELGIUM

## Abstract

Global collaboration continues to grow as a share of all scientific cooperation, measured as coauthorships of peer-reviewed, published papers. The percent of all scientific papers that are internationally coauthored has more than doubled in 20 years, and they account for all the growth in output among the scientifically advanced countries. Emerging countries, particularly China, have increased their participation in global science, in part by doubling their spending on R&D; they are increasingly likely to appear as partners on internationally coauthored scientific papers. Given the growth of connections at the international level, it is helpful to examine the phenomenon as a communications network and to consider the network as a new organization on the world stage that adds to and complements national systems. When examined as interconnections across the globe over two decades, a global network has grown denser but not more clustered, meaning there are many more connections but they are not grouping into exclusive ‘cliques’. This suggests that power relationships are not reproducing those of the political system. The network has features an open system, attracting productive scientists to participate in international projects. National governments could gain efficiencies and influence by developing policies and strategies designed to maximize network benefits—a model different from those designed for national systems.

## Introduction

Science has become increasingly collaborative and team-based [1–3], challenging governance mechanisms and evaluation processes [4–6]. A growing percentage of these collaborations happen at the international level [7–11]. In advanced countries, international cooperation represents all the growth in output [12, 13]. The percent of all scientific papers that are internationally coauthored has more than doubled in 20 years (Table 1), and many more people and countries are participating in international cooperation. Many more addresses are listed on papers with authors from at least two nations. Internationally coauthored papers are more highly cited than single-nation papers [14, 15] just as coauthored publications are more highly cited than single-authored papers [16].

The rise in scientific collaboration has attracted increasing interest among scholars. A literature has arisen, initially out of the biological/medical sciences, focusing on the “science of team science,” summarized by Stokols et al. [17], and discussed by the National Research Council of the U.S. National Academies [18]. This literature examines dynamics associated with cooperative research groups. Other research examines cases of international collaboration by field of discipline or topic, such as hypersonic flight [19], or Antarctic research [20, 21]. Other literature examines geographical participation in collaborative research, such as Taiwanese participation patterns [22], European patterns [23], European integration [24, 25], and Chinese participation [26]. Others examine collaboration in international relations contexts, such as Hollingsworth & Gear [27] or as part of political networks [28]. Efforts to map world scientific cooperation are in early stages, as seen in Boyack [29] and [30]. Still others examine patterns of R&D spending, noting that global spending on R&D has risen to over $1.5 trillion annually [11].

Of note in the literature has been a growing focus on developing countries and their ability to participate in scientific collaboration. UNESCO [31] reports that the rise in the number of internationally coauthored articles has happened along with a rise in scientific capacity among developing countries [32 33 34]. Much of this rise takes place through connections to developed countries, as scholars have observed for some time (see Allen, Piepmeier, and Cooney [35] already in the 1960s observing that international gatekeepers may have a central function in the network [36]). UNESCO further reports that, as scientific capacity has grown, the number of trained STEM workers (science, technology, engineering, and mathematics) is rising. Other research shows a rapid increase in student exchanges at the international level [37, 38]. These trends increase the capability and number of researchers available for collaborative research.

As international collaboration has grown, it is possible to argue that the shift towards the global challenges the relationship between science and the state. Collaboration has grown for reasons independent of the needs and policies of the state. Reasons for the growth of collaboration appear to be related more to factors endogenous to science, such as the location of equipment (such as telescopes or synchrotrons), need to access resources (such as location of soil or ice fields), and investment in and dispersion of talent. Exogenous factors have also changed, including reduced cost of travel. Notably, financial woes have put pressure on R&D spending in scientifically advanced countries—a factor which may be spurring part of the internationalization of collaboration as research leverage scarce funds. Other exogenous factors include the coordination needed to address global challenges such as climate change, access to water or international fishing resources, and control of infectious diseases such as Ebola [39]. As Bruno Strasser [40] points out, “National identity has linked science and state in subtle ways since the scientific revolution, but during the cold war, this relationship grew particularly strong as nation-states became the main patrons of scientific research….[during which] science…played an essential role in the construction of national identities…” (p. 165–66). These forces have waned. Since the end of the Cold War in 1990, the relationship between science funding and national identity has shifted considerably [27], as has the growth of international collaboration, but the direction of causality is unclear and remains a subject for further study.

As political and economic shifts have occurred over the past three decades, we see the growth of international collaboration as decoupling from the goals of national science policies. Public investment in science and technology is often justified on the basis of a contribution to national economic growth [41, 42]. Science policies often have the goal of promoting national scientific excellence [43] and economic competitiveness [44], but with a national flavor. This is partly due to the fact that public spending is accountable to citizens, thus science has retained a national character, even as the sources of knowledge become more dispersed. Public spending is often tied to national missions such as energy research, health care advances, agricultural productivity, and so on. National governments continue to be accountable to the public for serving these missions, no matter where the underlying science is sourced. Ironically, the growth of international collaboration does not threaten national science–in fact, it appears to enhance its quality. International collaboration does, however, challenge the evaluation of outcomes and accounting for capacity building. Geographical dispersion may reduce some local benefits that once accrued around capacity building and diffusion, but again, this is understudied. This paper describes the growth of global networks of science and discusses a possible approach to evaluation and accountability based in network analysis. We add new data to enhance the understanding of the global system, and we explore the influence of the global level on science policy.

## Methodology and Data

A commonly used measure of international collaboration in science is to count coauthorships in publications that appear in refereed journals. The authors collected citable publications (articles, reviews, and letters) for 2011 from the Science Citation Index (SCI) (CD-Rom version, the same data source used by the U.S. National Science Board [44]. (The data are not from SCI-Expanded, but from the CD-Rom version.) Coauthorship events are the most formal and widely-used indicator of international collaboration. From a methodological perspective, coauthorship counts have the advantage of being reproducible over time and traceable year-on-year—a method the authors have used in the past [45, 46] and present here to analyze changes and trends. We acknowledge that this mode of counting is only one among several possible measures of collaboration, and that scientific collaboration may lead to a number of outcomes of which the coauthored paper is only one [47, 48]. Conversely, we acknowledge that coauthorship in itself does not mean that collaboration has occurred [49]. The measure is widely used in practice [50], upheld by theory: Price [51], Crane [52], Narin [53] assert that the submission of manuscripts containing new knowledge claims is the crucial outcome of science, representing findings that the authors collectively are willing to claim as notable. The claim of authorship serves as a socio-cognitive filter on the multitude of relations in the social context of discovery [50].

The address lines in records of scientific publications list the names of contributing institutions and countries. The counts of country names and their interconnections (based on addresses attributed to authors) are made by examining all the records in the abstracting database for 2011. This data enables us to count coauthors from a single country (domestic) or different countries (international). (Papers with a single institutional address are counted in the asymmetrical matrix of document versus countries but not in the symmetrical matrix of international collaborations.) The first step is to make an asymmetrical (two-mode) matrix of documents versus countries. This matrix can be multiplied with its transposed (countries versus documents) for generating a symmetrical co-occurrence matrix (among 201 countries). This was done using Pajek. The asymmetrical matrix was first binarized in order to avoid double counting in the case of more than a single address from a country attributed to a paper. Cosine similarity is based on the asymmetrical matrix. We count numbers of documents; in the case of two authors from the US and three from China, this is counted as a single international coauthorship relation (and not 2 x 3 = 6 affiliations).

## Descriptive Statistics

Table 1 shows the number of records collected for 1990, 2000, 2008, and 2011. In raw counts, the number of records in the database increased from 508,941 in 1990 to 778,988 in 2011. Actual increase due to growth is about 46 percent over the period studied. Notably for our inquiry, the number of authors in that set has risen by 60 percent, much faster than would be suggested by the 15 percent rise in the number of journals counted, suggesting that there are more coauthors-per-paper; further corroborating this observation is that the addresses in the file increased at nearly the same rate as authors, from 908,783 in 1990 to 2,101,384 in 2011, an increase of 57 percent over the time span studied. Of these addresses, 2,099,104 or 99.9 percent are included in our analysis of institutional addresses. (The other 2,370 addresses were invalid.) Within the data set, internationally coauthored records increased by 73 percent between 1990 and 2011 (from 51,601 to 193,216 records). In 1990, internationally coauthored records had an average of 2.9 authors; by 2011, this same set had an average of 4.3 authors. The final line in Table 1 shows the percent of internationally coauthored records in the data set increasing from 10 to nearly 25 percent of all publications in those years.

**Table 1:** Data on international collaborative research papers, 1990, 2000, 2005, and 2011.

	1990	2000	2005	2011
Relevant records	508,941	623,111	734,750	787,001
Number of journals	3,192	3,745	3,722	3,744
Number of authors	1,866,821	3,060,436	3,301,251	4,660,500
Addresses in the file	908,783	1,432,401	1,696,042	2,101,384
Internationally coauthored records	51,601	121,432	171,402	193,216
international addresses	147,411	398,503	618,928	825,664
% internationally coauthored records	10.14	19.49	23.33	24.55

**Fig 1:**
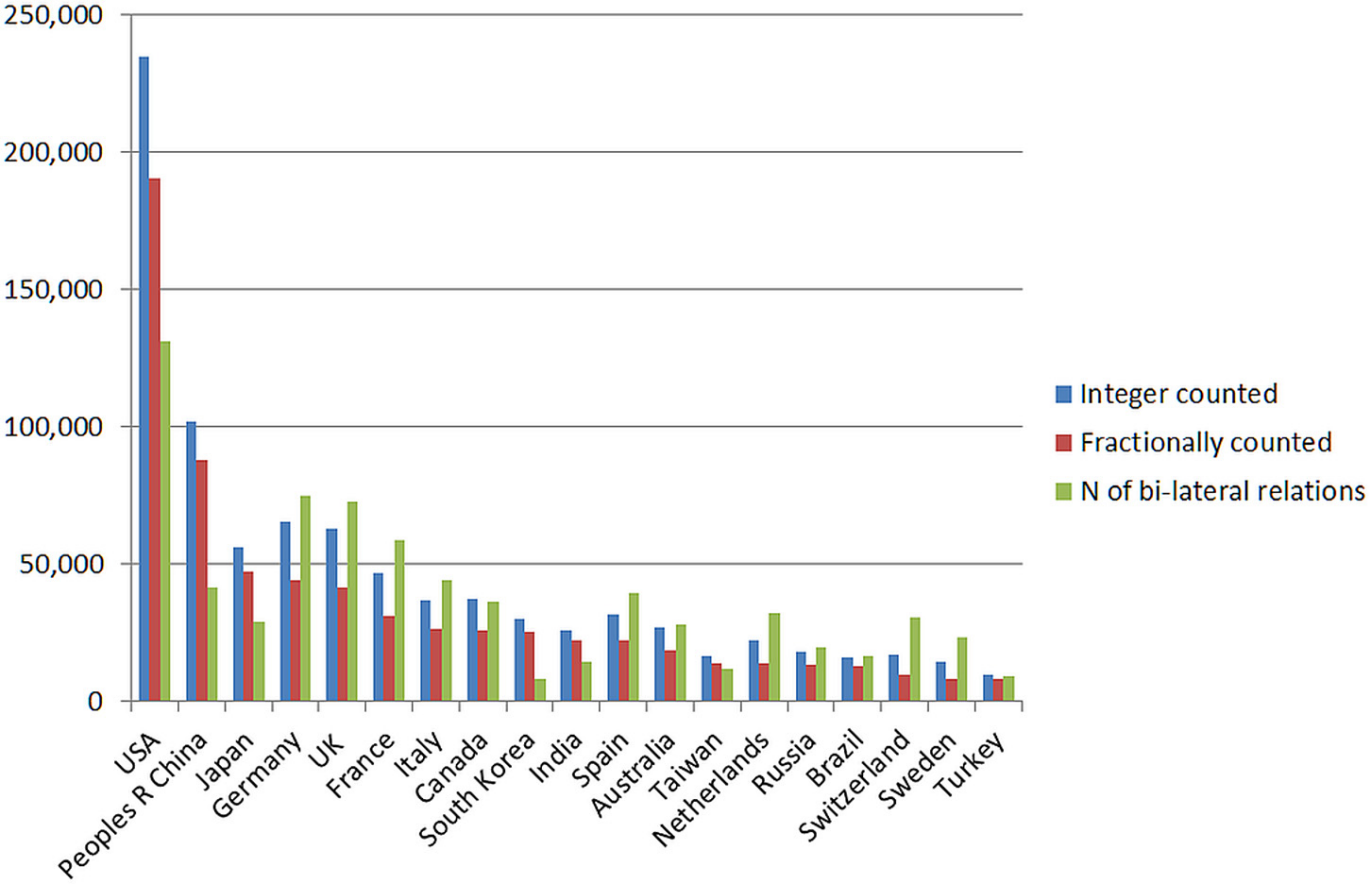
Counts of articles, reviews, and letters, 2011, integer and fractional counts (SCI data); the number of bi-lateral relations is based on the margin totals of the matrix of international collaborations.

Fig 1 illustrates the coauthorship counts for those countries producing the largest numbers of articles (count limited to 20). The counts are made in three ways, 1) fractional counts (where each address gets a percentage share of the relevant record); 2) integer counts (where each address gets a count of one); and 3) the number of international collaborations as the number of bilateral relations counted in the co-occurrence matrix (e.g., a paper with three US addresses, two French and one Italian is counted as one US-FR, one US-Italy, and one FR-Italy). Notably, the European countries show higher rates of international collaboration than the three other leading producers (USA, China, and Japan). This suggests that Europe has had greater internal integration compared to the rest of the world. We did not further subdivide international collaborations for EU countries into within-EU and other collaborations because such further analysis would lead us astray from the argument in this study given the changing borders of the EU during the period under study [54–56]. This may in part be due to policy requiring participation by at least three European Union countries in publicly-funded research projects. Using this third indicator, a single paper would then be counted at least three times in terms of international collaborations.

## Network Analysis

Network analysis has been shown to be useful in examining communication processes such as scientific collaborations [57]. Network analysis has been increasingly used to analyze international collaboration in science, as seen in Wagner & Leydesdorff [45]; Pan et al. [58]; and Adams et al. [59]. Coauthorships can be considered as a proxy for collaboration between scientists across institutions and coauthorship occurrences can be mapped as links in a communications network. Links between authors evidenced by coauthorships can be aggregated and presented as a formal communications network, with connections revealing patterns of relationships and knowledge pathways that may be enhancing research activity and resource opportunities. Jones, Hesterly, and Borgatti [60] proposed that networks arise in conditions of task complexity, which appears to be a feature of some kinds of scientific research [61].

We conducted network analyses to examine whether the network changed as the number of authors and paper increased. We expected to find that the increase in the number of participating nations (and the addition of many more addresses, Table 1) has had the effect of increasing the density of the network. We expected to find a lower clustering coefficient—meaning that many links that could be made are made. We further expected high betweenness measures–meaning that some countries have greater visibility and power within the network to attract others into collaborative relationships. Finally, we expected to find a tight core group—meaning a group of frequently interacting countries—with less developed countries falling into a periphery around a core, similar to earlier findings in a study of the global network [45].

Network measures are made in two ways: 1) a ‘raw’ set of connections among all coauthoring scientists, aggregated at the national level; 2) a cosine-normalized network, also aggregated at the national level. Literature has discussed the proper normalization measure: It has been suggested and is now widely accepted that the cosine instead of the Pearson correlation is the proper measure for normalization because of the non-normal distributions [62–64, 29]. The cosine normalizes the data, but without the assumption of normality in the data; the consequent vector space model creates data that can be visualized as a three dimensional ‘universe’ of connections. The resulting networks can be analyzed and the statistical features can be interpreted. Since the cosine runs from zero to one, a small number of relations can be expected to generate a cosine larger than zero. We considered cosine >0.01 as a relevant threshold for discarding incidental variation. When the data are normalized for the size of participating countries using the cosine, the *k*-core reveals a latent structure in the network. The core component in this case is the *k*-core, a subset of the network where each node has at least *k* neighbors. The *k* is a degree measure determined from an analysis based upon the size of the entire network.

To examine the network structure, we drew from the cosine normalized data which normalizes for the size of participants that includes large and small countries. The data were imported into UCINet and Pajek software to create networks and gather statistical signatures. Table 2 shows some input data and network results.

**Table 2:** Network statistics for the global network of science, 1990, 2000, 2005, 2011.

**Global network statistics**	1990	2000	2005	2011
**Number of countries**	172	192	194	201
**Number of coauthor relationships (links)**	1926	3537	9400	12029
**Size of k-core component**	35	53	64	114
**Density**	.13	.19	.25	.30
**Average degree**	22.4	36.9	48.6	119.7
**Average distance**	2	1.9	1.8	1.7
**Diameter**	3	3	3	3
**Graph betweeness**	.26	.16	.14	.1
**Average clustering coefficient**	.78	.79	.79	.79

As shown in Table 2, the greatest change from earlier assessments [45, 46] can be observed in the size of the k-core component—the dense center of the network which was limited to 35 countries in 1990 just as the Soviet Union was dissolving and East and West Germany were reintegrating. Table 2 shows the number of countries in the core increase rapidly to 64 in 2005 and then to 114 nations in 2011. This growth suggests that most nations have scientists who are participating actively in international collaborative networks. It further suggests that the network is not recreating political or geographic structures. A second change is the increased density of the network from .13 to .30, suggesting that many more connections have been forged by more partners. This finding tracks with the numerical growth in the number of coauthor relationships (links) in Table 2; the increase in links is disproportionately large compared to the growth in the number of addresses in the file shown in Table 1.

The average degree indicates the spread of influence of countries across the network; the average distance has dropped to lower than two—meaning that if one were to conduct collective search (for friends-of-friends) one can potentially reach others by just a few ‘handshakes’ across the network. The diameter of the network remains at three, meaning that the network of nations can be traversed in three steps from any node on one edge to any node on the other edge. Against expectation, the average betweenness among nations has dropped from .26 to .10 suggesting that fewer nodes dominate the network, or, in other words, power is more diffused throughout the network in 2011 than was the case in 1990. New entrants are not clustering around the scientific ‘leaders’. This can also be interpreted as representing a more open network than was found in 1990. The average clustering coefficient rose slightly, also against expectation, further bolstering the observation on openness.

The network analysis shows increasing density. The measures defied expectations around a dense core group—the core group grew membership quite considerably, with many developing countries also joining the core group, meaning that new members find it relatively easy to join. Moreover, the network defied expectations in the average degree and betweenness measures, where we expected to see greater recreation of political boundaries and power relationships. Instead, the average degree suggests that power is being dispersed throughout the network rather than concentrating around large players. The drop in betweenness measures suggests that many nodes operate effectively in the network, and that influence is not accruing to centralized nodes.

## Growth in Foreign S&T Capacity as a Contributing Factor

The growth in the network may be explained in part by an increase in S&T capacity in many countries over the past 30 years. Capacity building has enabled researchers in many more countries to collaborate [64]. Over the past several decades, science has benefitted from striking shifts in spending, output, capacity, and international participation to include more nations. For example, in 1990, six countries were responsible for more than 90 percent of public spending on research and development (R&D); by 2008, 13 countries were responsible for 90 percent of public spending, not including the spending by the European Commission [31]. During the same time, developing countries doubled their R&D spending [31]. UNESCO reports that the number of researchers claimed by countries around the world rose from fewer than 5 million in 2000, to more than 7 million by 2007. A review of Thomson-Reuter’s Web of Science shows that in the 2000s, many more nations actively participated in research and research collaboration than was the case in the previous decade. The number of countries whose addresses appear in the network has grown to 201 countries up from 172 countries in 1990. (At the time, the Soviet Union was counted as a single nation so some of this growth is due to the breakup of the Soviet Union.) Even famously isolated North Korea can be found among the addresses of global collaborators.

Among the nations with the greatest increases in R&D spending in the 2000s, OECD reports that China became the second largest R&D performer after the United States in gross terms by 2011 [11]. China has a lower rate of R&D spending as a percentage of gross domestic product/ gross domestic expenditure on research and development (GDP/GERD) than many other countries, but with an upward trajectory. Similarly, in citation counts, Chinese papers are less cited than those from other countries, even when output is equivalent, but quality measures of Chinese publications also have an upward trajectory [65]. Other countries that made significant gains in R&D spending as a percent of GDP in the 2000s were South Korea, Denmark, Slovenia, Estonia, Czech Republic, and Portugal [66].

In terms of scientific output, a similar expansion of the global system can be seen. Adams [12] shows that China, Brazil, India, and South Korea increased their scientific output 20-fold over 30 years from 1981 to 2012, increasing the number of papers annually from 15,000 to more than 300,000. Leydesdorff and Wagner [46] show that China and South Korea have been growing their S&T output at spectacular rates over the past two decades. In an analysis of global output in the 2000s, Horlings and Van den Besselaar [67] show that the lower income, smaller economies grew their scientific output faster than larger systems. For most countries, collaborative research (represented by coauthorships) is becoming the norm. As noted above, for the scientifically advanced nations, the internationally coauthored articles account for almost all the growth; the same study find that, for developing countries, the majority of growth is still domestic output [12].

Because of its large size, in sheer numbers, the United States is the largest contributor of partners for international collaborations, although in percentage terms, it is among the lowest collaborator. U.S.-based scientists are more likely to find a domestic collaborator than are researchers from the smaller, scientifically advanced countries, such as Switzerland or the Netherlands. As a result of the number of U.S. researchers, U.S.-based authors appeared on 43 percent of the world's internationally coauthored articles in 2008 [44], and appear as directly linked to most of the countries and indirect links to all countries in the global network.

## The Dynamics of the Global Network

In earlier research, we suggested that “international collaboration in science can be considered as a communications network that is difference from national systems and has its own internal dynamics” [46]. The network data further corroborates this expectation, particularly in that political or geographic patterns cannot be seen in the data. The collaborations represent self-organizing phenomena, which may be influenced by features embedded in disciplines, such as equipment or resources required. These will be explored in a future article.

The international collaborative network has been growing over the past two decades which may be taking on organizational features. The structure of the network appears to be robust—meaning that individual nodes may be exchanged, removed, added, or renewed without altering the macro-behavior of the network. Monge & Contractor [57] find that the macro-behavior of a network is not the result of the micro-features or motivations of the agents. The formation and persistence of structure becomes the equivalent of an organization. The network provides attractive, resource-based opportunity to participants. The international level offers benefits that outweigh the transaction costs of working with people who are geographically remote.

**Fig 2:**
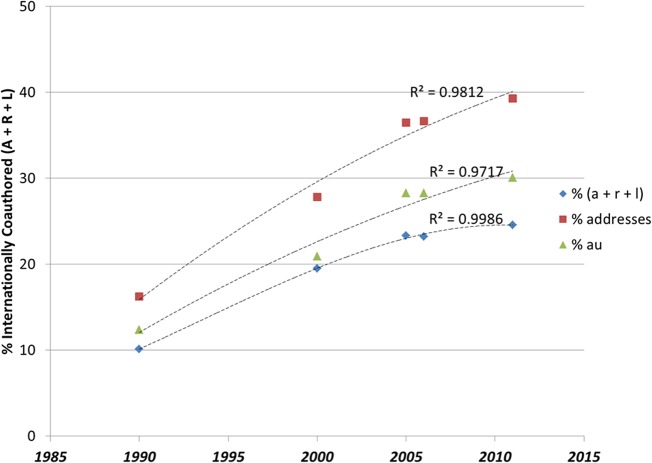
Growth of internationally coauthored records and addresses, 1990, 2000, 2005, and 2011.

Fig 2 shows the growth in the number of addresses of internationally collaborating authors from 1990 suggesting that the growth of the network is partly accounted for by new entrants (new addresses and new nations). This finding indicates that the global network is attracting new members and may be operating as an open system. The structure suggests that new entrants are able to find collaborators without having to pass first through a core of highly powerful (or central) nodes. Indeed, unique addresses in the file have more than doubled in number, so new institutions are involved in publishing in source journals at the international level than in earlier years. (These data are based on the CD-Rom version that selects 3,744 core journals from the Web-of-Science set; for the purpose of policy analysis (S&E Indicators of the NSB).) The number of authors for all records has tripled since 1990, indicating an expansion of participants in the network. Some expansion can be accounted for in the growth of the database, but that alone cannot explain the rapid growth: a net number of new participants have joined the network. Participation at the global level differs by country, as can be seen by examining the graphs at www.leydesdorff.net/intcoll/intcoll.htm. In 2009, the National Science Foundation published a report showing that international coauthorship trends for China, South Korea, and Taiwan have remained flat while other nations have become more internationally linked [44].

## National and Global Networks

The global network is attracting highly productive researchers and rewarding them with increased attention. The more elite the scientist, the more likely it is that he or she is working at the international level. We can assume that national priorities influence the choice of research topics, but how does the global network influence researchers’ choices about partners and topics? This question is difficult to answer through a survey method, since individual practitioners are likely unaware of the influence of the system on their choices.

To test for the influences of the global network on the national research effort, we compare the international network to national networks in terms of distributions of institutional addresses. Which of these two distributions can be seen as a better predictor of the other, and in which nations? The two vectors correlate significantly (*p* < .01) across the file, but they are also different: Pearson’s *r* = 0.840 and Spearman’s ρ = 0.653. Our interest, however, is in the difference: to what extent can the layer of internationally coauthored papers be considered a predictor of the domestic publication pattern at the level of a nation, or is the reverse arrow prevalent? The more internationally connected the scientific workforce of a nation, the more likely it is that the national agenda is being set *de facto* at the global level.

In order to conduct the test, the data were further refined to select only institutional addresses with more than 100 citable items in the file. This reduced the number of organizations to 2,420 worldwide, which was 74.7 percent of the set of international and domestic addresses. The file also contains the city-addresses and it would be possible to disaggregate, for example, the “Chinese Academy of Science” into its different affiliations, but in this study we are not specifically interested in the domestic networks per se (Leydesdorff & Persson, 2010), but rather in the relationship at the international level, and in the share of activities between domestic and international addresses. The Kullback-Leibler divergence provides an asymmetrical measure that allows us to specify the extent to which the one distribution can be considered a predictor of the other asymmetrically. This information measure can be derived from Shannon’s entropy measures [68][69], and informs us in bits of information about the difference between two distributions. In formula format, the equation can be written as follows:

Iq|p=∑iqi*log2(qipi) (1)

In Eq 1, *I*_q|p_ is the amount of expected information in the *a posteriori* one Σ_i_ *q*_i_ given the *a priori* probability distribution Σ_i_ *p*_i_. If the two distributions are similar, knowledge about the one distribution perfectly predicts the other and no uncertainty is generated. However, if the *a posterior* probability distribution is different from the *a priori* one, *I*_q|p_ is necessarily larger than zero ([69], p. 59 ff). The better predictor is the one which generates the least information upon the measurement.

We applied this measure to test the domestic distribution of publications in a nation (across institutional addresses) as a predictor of the distribution of internationally coauthored publications (at the level of nations), and *vice versa*. In total, 2,420 institutional addresses in 79 nations pass the threshold of more than 100 citable items in the file and are included in the analysis, but, of these, 18 nations are represented with only a single institutional address with more than 100 citable items. The case of a single unit of analysis nullifies this test because the distribution no longer contains uncertainty. As a result, the authors removed these countries from the file: only (79–18 =) 61 countries are used. (Changing this threshold of 100 citable items and more than a single institution would primarily affect the inclusion of marginal nations but much less the results of this analysis.)

The countries and states are evaluated on the question of whether the uncertainty in the international distribution given the national one—or in formula format: *I*(international|domestic)—is larger or smaller than *I*(domestic|international). If the latter is *smaller* than the former, the distribution of internationally coauthored publications is a better predictor of the distribution (in this case, *a priori*) of the nationally (co-)authored publications (*a posteriori*) than *vice versa*. In the maps, the political units in which the international dimension provides the better predictor are colored green, the national ones are colored red. The values of Kullback & Leibler’s [68] divergence measure are quantitative (in bits of information), but normalization issues are involved when one wishes to compare across countries or states. A larger number of institutional addresses in a country (e.g., the USA) may lead to a larger chance that Shannon-type information is generated in the comparison. In the case of a country with only two addresses, for example, changing a single publication may shift the results. These normalization issues would lead us beyond the scope of the current study, and we therefore decided to use the measure mainly as a binary test on whether the domestic or the international distribution provides the better prediction. In the case of equality of the two predictions, we count this conservatively as a “domestically” driven unit of analysis (although this case did not occur).

**Fig 3:**
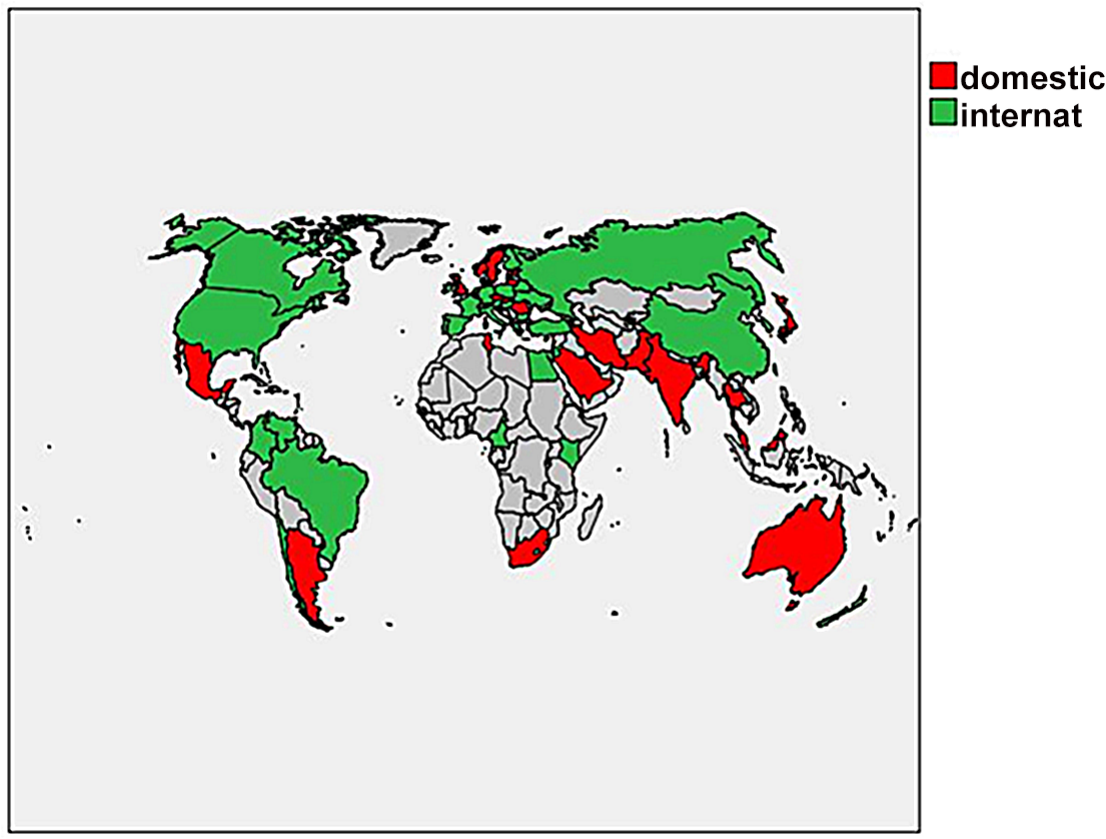
Map of 61 countries in terms of domestic distribution (red) or international distribution (green) providing the better indicator. Source: Authors using SPSS v.21 (under the campus license of the university.)

Of the 61 countries, the domestic pattern provides the better predictor in 27 cases; in the remaining 34 countries, the international pattern provides the better predictor. The results are visualized in Fig 3. (A separate article is being prepared that shows the influence of the international or national networks at the level of the U.S. states.)

The map distinguishes between those countries where the international distribution is a better predictor of the national distribution than vice versa: in the Americas these countries are the United States, Canada, Brazil, Chile, and Columbia. The reverse is shown for Mexico and Argentina. In Europe, the United Kingdom, and the Nordic countries show a stronger domestic pattern, while many other EU countries plus Russia show the distribution of international papers over institutional addresses as the better predictor. In Asia, the international indicator prevails for China and South Korea, whereas for Japan the domestic distribution provides the better predictor.

The nations do not align by the extent to which they are internationally connected as we expected. Instead, a geographic explanation appears to be more explanatory. Two types of nations are domestically driven: one is the nation that is geographically isolated (e.g., Japan and Australia) or underdeveloped (e.g., India and Egypt), and another is the nation with a strong national identity and a history of science tied to national development such as the UK and Scandinavian countries. These latter countries are ones that, while they participate in the European Union’s framework program, still maintain their own more strongly organized national institutions and policies.

In summary, the international and national networks may be shaping each other in a process of co-evolution between the national institutional structure and the global network. The relative influences of national and international networks appear to vary among nations. Globalization and internationalization can first be considered as a tendency, but in more than half of the countries, the international network has become the better predictor of the national participation at the global level than *vice versa*. In other cases, national patterns of collaboration still prevail.

## Implications for Research Policy

As international collaboration in science has grown, the role of the state in directing investment comes into question as does the fate of local research. As we have shown, international collaboration in science has risen dramatically over the past three decades, changing the landscape for scientific research in favor of global networks. As a share of all scientific articles indexed in the Web of Science, internationally coauthored articles rose from 10 percent in 1990 to nearly 25 percent of indexed articles in 2011. This suggests that growth of science is occurring to a disproportionate extent at the global level, which may be attracting more prominent scholars to work together across national borders, and drawing away top thinkers from focusing on local needs [Barnard et al., 2012]. Moreover, the shift towards global collaboration may be presenting competition for rapid assimilation of the results of research locally. Leigh et al. noted that local communities fear they “have been losing control over their destinies as the nation has increasingly become tied to global rather than national and local forces….” ([70], p. 4).

We asked whether the global network is influencing the direction of scientific investment at the national, regional, and local levels. While more research is needed, the tests conducted suggest that the global system is highly influential for some countries. If current trends continue—ones where highly elite scientists work at the global level, and where developing countries increasingly participate—the challenges associated with the global network may be in the gaps created at the local levels. Distributed research that favors the use of the most efficient producer may enhance overall outcomes, but it can come at the expense of capacity building at local levels.

National leadership in science has been characterized as having historical patterns tied to political, economic, and military power. Recently, Hollingsworth and Gear [27] tied scientific leadership to economic, political, and military hegemony over 275 years. In an argument similar to that offered by Bernal [71] and Ben-David [72], they trace scientific leadership from France to Germany in the 19^th^ century, from Germany to Britain in the early 20^th^ century, and from Britain to the United States in the mid-20^th^ century, claiming that scientific leadership is closely aligned to hegemony with multi-factored causes. The current growth of international collaborations appears not to reinforce these patterns and puts into question the relationship between science and the state.

The growth of the global network is an emerging organization added to (and possibly superseding) the national model. The organization may be more open to new members, since greater density of the network and the lowered betweenness measures suggest that fewer of the communications pass through the leading nodes or countries. This may mean reduced influence for advanced countries, and shifting of power to some ‘peripheral’ nodes. Glänzel et al. [14] found that international cooperation is particularly advantageous for less advanced countries; network participation should enhance that advantage because it enables efficient collective search. The overall system (global and national) may become more productive and efficient, but at the expense of national visibility and local connectivity. It is difficult to measure this kind of dynamic, and yet, the growth of the global network suggests that better understanding of these knowledge flows may be very important to knowledge appropriability in the future. More research is needed to understand this set of linkages and their implications.

The active and robust global network is proof of its own usefulness. Researchers gain enough benefit from it that they are willing to extend the extra time and effort to maintain long-distance communications. Should capacity continue to grow in more places around the globe, one can expect to see more ‘nodes’ join the network. The global network is arguably now a more stable system that serves as a source of vitality and direction to R&D at all lower levels. Our results show that the global level appears to be a strong influence on U.S. scientific research, for example. The international influences are seen by the U.S. National Academy of Science [73] as providing a “global platform” for research that is claiming the attention of the growing number of the most elite U.S.-based scientific researchers. Knowledge is at least shared with foreign partners if not jointly developed.

The global network presents opportunities for science policy-makers to seek efficiencies that were not available when a few nations dominated science. With improved scanning of research and more effective communications, it may be possible to leverage foreign research, data, equipment, and know-how to aid U.S. science, thereby freeing up researchers to explore more groundbreaking work. Just as economic impact statements are requested now, it may be possible to ask grant seekers to identify possibilities for efficiency gains through international collaboration, and then provide the financial and policy supports to integrate knowledge from abroad.

This dynamic system, operating orthogonally to national systems, is increasingly difficult to influence and even less amenable to governance as it grows. This does not mean that nations must build an international governance mechanism, but that they must learn to manage and benefit from a network. Networks operate by reciprocity, exchange, incentives, trust, and openness, so explicit policies of support for complementary links; for ease of gaining visas for people, equipment, and specimens; for incentives to collaborate in critical areas; of smart specialization where needed, are likely to boost the ability of the U.S. to benefit far more from the global system than it is now. At the global level, no ‘ministry of science’ issues calls for proposals: participants seek to access the enhanced opportunity for knowledge creation. Science policy-makers can take advantage of the global system by seeking to increase access to, leverage of, and support for the collaborative projects that emerge.
